# Research Progress on Structure and Anti-Gynecological Malignant Tumor of Shikonin

**DOI:** 10.3389/fchem.2022.935894

**Published:** 2022-07-08

**Authors:** Li-Na Ke, Ling-Qi Kong, Huan-Huan Xu, Qin-Hua Chen, Yun Dong, Bin Li, Xiao-Hua Zeng, Hong-Mei Wang

**Affiliations:** ^1^ Sinopharm Dongfeng General Hospital, Hubei University of Medicine, Shiyan, China; ^2^ Hubei Key Laboratory of Wudang Local Chinese Medicine Research, School of Pharmaceutical Sciences, Hubei University of Medicine, Shiyan, China; ^3^ Shenzhen Baoan Authentic TCM Therapy Hospital, Shenzhen, China

**Keywords:** shikonin, anti-cancer, gynecological malignancy, structural modification, review

## Abstract

Gynecological malignancy seriously threatens the physical and mental health of women. Shikonin is a naphthoquinone compound with a variety of biological activities. Studies have shown that shikonin can inhibit cell proliferation, promote cell apoptosis and induce cell necrosis. And in recent years, shikonin are also being increasingly used for the study of gynecological malignant diseases. Therefore, we reviewed the mechanism of action and structure optimization of shikonin in gynecological malignant tumors, in order to provide some reference for further research and development of related drug.

## 1 Introduction

Lithospermum erythrorhizon Sieb. et Zucc. referred to as “Zicao” in Chinese and “Shikonin” in Japanese, is a perennial herbaceous plant of the purple grass family (Zhang et al., 2012). According to the Shennong Bencaojing (Shennong’s Classic of Materia Medica), shikonin has various functions of cooling blood, promoting blood circulation, detoxifying and penetrating rash (Guo et al., 2019). The 2020 edition of Chinese Pharmacopoeia also included Lithochroma from Xinjiang (*Arnebia euchroma* *(Royle) Johnst.*) and Lithochroma from Inner Mongolia (*ArnebiaguttataBunge.*) (Commission, 2020). Shikonin is a red natural compound with naphthoquinone structure, which exists in the roots of Zicao. It is not only a natural pigment, but also has high medicinal value, including antiviral, anti-inflammatory and anti-tumor effects (Miao et al., 2012; Moon et al., 2014; Han et al., 2021), especially the anti-gynecological malignancy effects have been proved by a large number of studies in recent years (Thakur et al., 2015; Shahsavari et al., 2018). Thus, this paper expounds the structure and anti-gynecological malignancy aspects of shikonin, in order to make wider use of shikonin in gynecological diseases.

## 2 Chemical Structures

**FIGURE 1:**
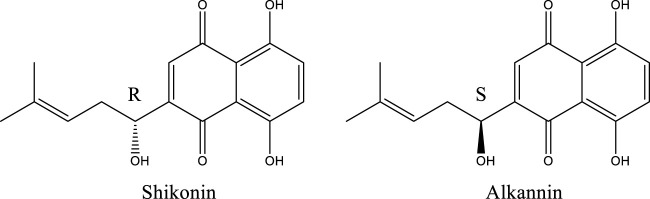
Chemical structures of shikonin and alkannin.

The basic parent nuclear structure of shikonin compound is 5, 8-dihydroxy-1, 4-naphthoquinone with isohexenyl side chains (Shukla et al., 2001). Because of their optical activity, shikonin compounds can be divided into two optical isomers: R-shikonin and S-alkannin (Figure 1). Since alkannin is roughly similar to shikonin in terms of distribution, but it has fewer plants than shikonin, most studies have focused on shikonin (Wang et al., 2022). Moreover, shikonin had more significant and extensive anti-tumor activities than alkannin. Therefore, the role of shikonin in gynecological malignant tumors was reviewed in this paper.

## 3 Anti-Gynecological Malignant Tumor Effects

**FIGURE 2:**
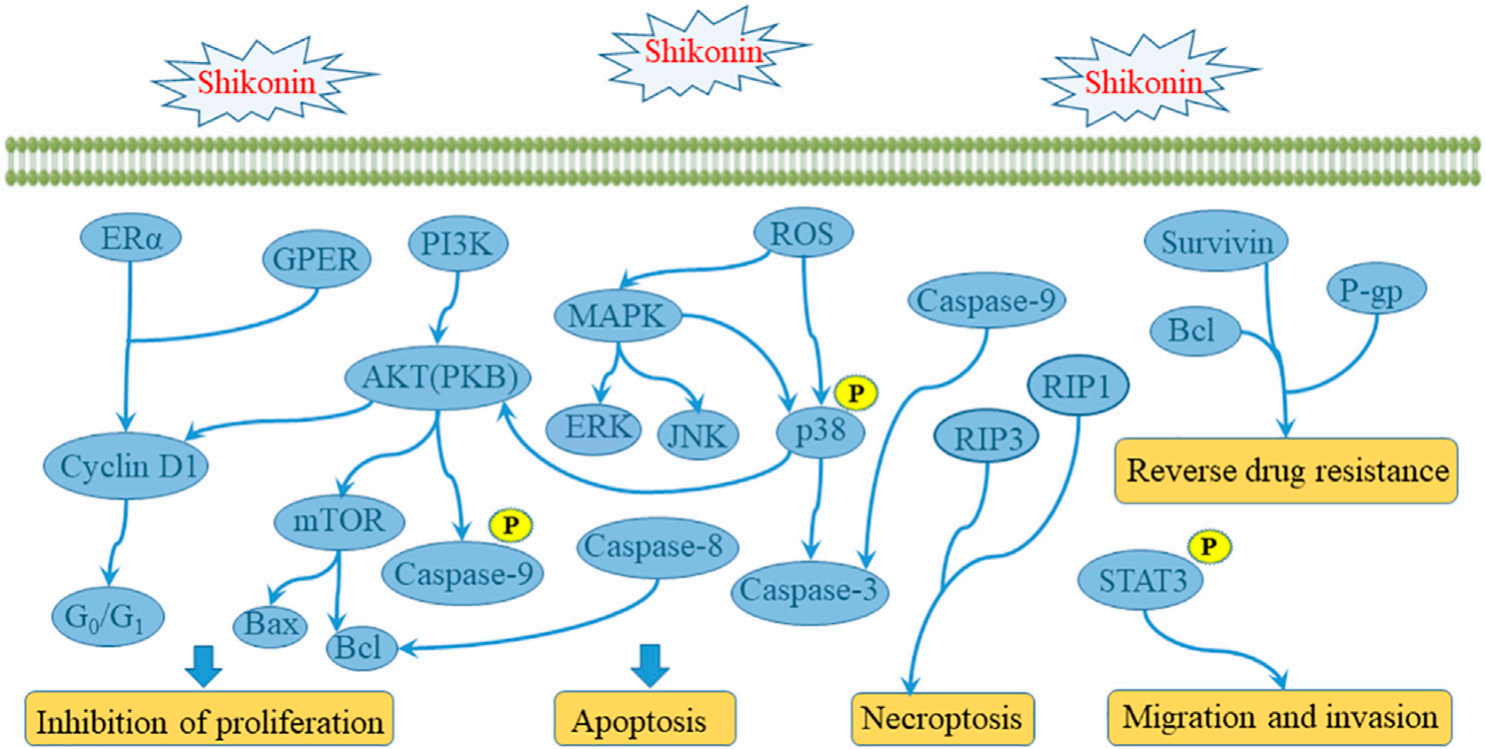
The signaling pathway of shikonin against gynecological malignant tumor.

Gynecological malignant tumors mainly include cervical cancer, endometrial cancer, ovarian cancer and choriocarcinoma. And in recent years, the incidence and mortality of gynecological malignant tumors are on the rise, which seriously endangering women’s life and health. Nowadays, the treatment methods of gynecological malignant tumors mainly with surgery, chemotherapy and radiotherapy, among which chemotherapy plays an important role in the treatment of gynecological malignant tumors. Therefore, we reviewed the effects of shikonin on gynecological malignancies (Figure 2).

### 3.1 Inhibit Cell Proliferation of Gynecological Malignant Tumor

#### 3.1.1 Estrogen Quasi Hormone Receptor Signaling Pathway

Estrogen can promote the development of female secondary sexual characteristics, establish and maintain reproductive function, in addition to regulating cell growth, division and differentiation and other physiological processes (Wang et al., 2022). The functions of estrogen are mainly realized through the combination of estrogen receptor (ER) in cells, which includes ERα, ERβ, and G protein coupling with estrogen receptor (GPER). Abnormal signal can lead to various diseases, such as endometrial cancer (EC), accounting for 20–30% of female reproductive tract malignancies. Nearly 80% of these were estrogen-dependent adenocarcinomas (Ⅰ type) (Jia et al., 2015). Found that shikonin inhibits the proliferation of endometrial cancer Ishikawa cells in a time-concentration dependent manner by inhibiting the expression of ERα in estrogen signaling pathway Huang et al. (2016).

#### 3.1.2 Block Cell Cycle Progression

Studies showed that shikonin can inhibit tumor proliferation by regulating the expression of cyclin-related proteins and blocking the progression of tumor cycle. Found that shikonin can block human cervical cancer Siha cells at G_0_/G_1_ stage, and the molecular mechanism of inducing G_0_/G_1_ block may be related to Cyclin D1 Yang et al. (2018). Found that shikonin can increase the proportion of G_0_/G_1_ cells in human choriocarcinoma drug-resistant cell line JAR/MTX cells, and decrease the proportion of S and G_2_/M cells Ma and Li (2008). Studies have found that shikonin can change the distribution of cell cycle in Hela cells of cervical cancer, and arrest cells in S phase Yu et al. (2013). Yin found that shikonin could block Ishikawa cells in G_0_/G_1_ stage of endometrial cancer, thus significantly inhibiting Ishikawa cell proliferation Yin (2016).

#### 3.1.3 Induction of Reactive Oxygen Species in Tumor Cells

Reactive oxygen species (ROS) are highly active and chemically active oxygen-containing substances generated by aerobic cells in the process of aerobic metabolism. ROS contains unpaired electrons and has a high reactive activity. Excessive accumulation of ROS under oxidative stress can produce cytotoxicity and cause damage to intracellular lipid, DNA, protein and other biological macromolecules, resulting in cell dysfunction and cell growth inhibition or even death (Zhu et al., 2016). Studies have found that shikonin inhibits the proliferation of human cervical cancer Hela cells in a dose-dependent manner, and the change of mitochondrial membrane potential and the generation of endogenous reactive oxygen species are one of the mechanisms of shikonin inhibiting the proliferation of human cervical cancer Hela cells (Yu et al., 2015).

### 3.2 Induced Apoptosis of Gynecological Malignant Tumor Cells

#### 3.2.1 Bax and Bcl-2 Mediated Signaling Pathway

Apoptosis is a kind of spontaneous and orderly cell death controlled by genes, which is one of the important mechanisms to maintain the stability of internal environment. The bcl-2 gene family is related to the regulation of apoptosis. Bcl-2 and Bax can inhibit apoptosis and induce apoptosis respectively, and which together operate as a cell life-death switch. Shikonin induces apoptosis of tumor cells by affecting the expression of pro-apoptotic protein Bax and inhibiting the expression of anti-apoptotic protein (bcl-2, Bcl-x, etc.) in the Bcl-2 gene family. Found that shikonin can effectively induce apoptosis and necrosis of transplanted tumor cells in nude mice with choriocarcinoma and significantly inhibit the secretion of human chorionic gonadotropin (β-HCG) through hematoxylin-eosin (HE) staining and immunohistochemistry Hu et al. (2004). The down-regulation of bcl-2 protein expression is an important mechanism of shikonin against choriocarcinoma effect. Studies have shown that shikonin can inhibit the proliferation and induce apoptosis of endometrial cancer Ishikawa cells (Yang et al., 2013) and human ovarian cancer SKOV-3 cells (Wang et al., 2016a) *in vitro*, and the mechanism may be related to up-regulation of Bax expression and down-regulation of Bcl-2 expression.

#### 3.2.2 Phosphatidylinositol Three Kinase/AKT Signaling Pathway

AKT is a serine or threonine kinase, also known as protein kinase B (PKB), which is an important apoptosis suppressor protein *in vivo*. Phosphatidylinositol three kinase (PI3K)/AKT signaling pathway has been confirmed to be involved in anti-apoptosis, promoting cell proliferation, migration and oncogenic transformation (Xie et al., 2017). Studies have shown that the expression level of Akt in cervical cancer is higher than that in normal cervical tissues and cervical intraepithelial neoplasia (CIN) tissues, and increases with the increase of CIN level (Liao et al., 2008). Proved that shikonin can induce apoptosis of endometrial cancer Ishikawa cells by inhibiting AKT phosphorylation in PI3K/AKT signaling pathway, up-regulating downstream target protein Bax and down-regulating bcl-2 protein expression Yu et al. (2014). Found that shikonin can inhibit the proliferation and promote apoptosis of isolated endometrial cancer HEC-1B cell by regulating PI3K/AKT apoptosis signaling pathway Xie and Xue (2017). Lu found that β-hydroxyisovalerylshikonin (β-HIVS) could induce apoptosis of Hela cells by inhibiting PI3K/AKT/mTOR signaling pathway and decreasing the expression levels of related downstream genes and proteins in this pathway Luo (2015). This signaling pathway is considered as the target of molecular targeted therapy of Traditional Chinese medicine and is expected to be a new approach in the treatment of cervical cancer.

#### 3.2.3 Mitogen-Activated Protein Kinases/Caspase/Reactive Oxygen Species Signaling Pathway

Mitogen-activated protein kinases (MAPKs) are serine or threonine protein kinases in cells, including ERKs, JNKs/SAPKs, P38, and ERK5/BMK, whose mediated signaling pathway is closely related to cell proliferation, differentiation, and apoptosis. Found that shikonin can induce the apoptosis of cisplatin resistant human ovarian cancer A2780 cells and activate JNK, P38, and ERK in a time-dependent manner, reducing the cytotoxicity of shikonin with the three specific inhibitors, thus demonstrating that shikonin can induce cell apoptosis through MAPK activation Shilnikova et al. (2018). Cysteinyl aspartate specific proteinase (caspase) is a protease that is closely related to programmed cell death and inflammation. Studies found that the apoptosis of human choriocarcinoma JEG-3 cells after treatment with shikonin was obvious. Western blot results showed that the proteins caspase-3, ERK and JNK of JEG-3 cells were activated. And the apoptosis of JEG-3 cells induced by shikonin was speculated to be related to caspase-3 pathway and MAPK pathway Huang et al. (2009). The generation of intracellular ROS is closely associated with cell apoptosis (Circu et al., 2009). Found that shikonin induced Hela cell apoptosis, resulting in the generation of ROS and significantly increased the expression of phosphorylated p38. The application of ROS scaver (NAC) and p38 inhibitor (SB203580) could significantly reduce the growth inhibition and apoptosis rate of Hela cells treated with shikonin Zhang et al. (2011). These results indicate that ROS/p38 signaling pathway is involved in the shikonin-mediated apoptosis.

### 3.3 Inhibit the Migration and Invasion of Gynecological Malignant Tumor Cells

#### 3.3.1 Epithelial-Mesenchymal Transformation

Epithelial-mesenchymal transformation (EMT) is characteristic of aggressive tumors and is characterized by decreased epithelial (E-) cadherin expression and increased neural (N-) cadherin expression, which contribute to stromal cell adhesion and enhance motility and invasion of cancer cells (Christiansen and Rajasekaran, 2006). Showed that shikonin could reduce the migration ability of A2780 cisplatin-resistant cells by weakening the EMT process (up-regulating EC and down-regulating NC) Shilnikova et al. (2018).

#### 3.3.2 STAT3 Signaling Pathway

STAT3, the most common and easily activated member of the STAT family in human malignant tumors, mainly mediates intercellular or extracellular signal transduction to intracellular, and which plays an important role in tumor genesis and malignant transformation. Found that shikonin could significantly inhibit the migration and invasion ability of human choriocarcinoma JEG-3 cells Wang et al. (2016b). The experiments showed that shikonin could reduce phosphorylated STAT3 protein and mRNA expression. In conclusion, the mechanism of shikonin inhibiting the migration and invasion of JEG-3 cells may be associated with the inhibition of STAT3 signaling pathway.

### 3.4 Inducing Necroptosis of Gynecological Malignant Tumor Cells

Necroptosis belongs to programmed cell death and has a complex relationship with necrosis and apoptosis. However, it is widely believed that necroptosis can be used as a substitute for apoptosis. When apoptosis fails, necroptosis will be activated as an “automatic failure prevention mechanism”, which may provide a new research direction for tumor therapy that is resistant to classical apoptotic pathways. Found that human ovarian cancer SKOV3 and A2780 cells showed significant proliferation inhibition after treatment with shikonin Feng et al. (2019). However, there was no significant change in apoptosis rate, but the proportion of necrosis increased significantly. Hoechst 33,342 and PI staining showed no obvious apoptotic characteristics, but Western Blot showed that the expressions of necroptosis pathway-related proteins RIP1 and RIP3 were significantly up-regulation.

### 3.5 Increasing the Sensitivity of Gynecological Malignant Tumor Cells to Chemoradiotherapy

In addition to killing tumor cells alone, shikonin can also increase the sensitivity of tumor cells to radiotherapy and chemotherapy. Found that shikonin can enhance the radiosensitivity of human ovarian cancer cell line SKOV3, and the mechanism may be related to the inhibition of PI3K/AKT signaling pathway and the change of cell cycle distribution Fan et al. (2019). Found that the inhibition effect of shikonin combined with cisplatin on cervical cancer Hela cells was significantly higher than that of cisplatin alone Du et al. (2018). Found that β-hydroxyisovalerylshikonin (β-HIVS) had synergistic sensitization effect on cisplatin within a certain concentration range, and the synergistic effect became more significant with the increase of its concentration Zhang et al. (2014).

### 3.6 Reverse Drug Resistance of Gynecological Malignant Tumor Cells

Chemotherapy is one of the three treatment methods for cancer, which can effectively control the growth, diffusion and metastasis of tumors, especially for some highly sensitive gynecological malignant tumors after surgery, it can achieve cure effect. Chemotherapeutic resistance is one of the main causes of tumor recurrence and treatment failure, which involves the overexpression of p-glycoprotein (P-gp), multi-drug resistant associate protein (MRP), glutathione-S-transferases (GSTs) and other mechanisms. P-gp is a transmembrane glycoprotein encoded by multidrug resistance gene MDR1, which is an ATP-dependent drug efflux pump. P-gp can pump intracellular drugs out of cells in the presence of ATP, reducing intracellular drug concentration and leading to cell resistance. GSTs is one of the most important phase II metabolic enzymes during biotransformation *in vivo*, and which also is the main detoxification system for cell anti-injury and anti-cancer transformation. Moreover, the change of GSTs expression level may be related to chemotherapy resistance of tumor. Studies (Yu and Li, 2007; Chen, 2009; Ren, 2009) found that shikonin can promote the apoptosis of choriocarcinoma JAR and methotrexate-resistant human choriocarcinoma JAR cells (JAR/MTX cells), cause the cell cycle arrest of JAR/MTX cells, and reverse the resistance of JAR/MTX cells to MTX. The mechanism is realized by down-regulating the expression of apoptosis suppressor genes Bcl-2 and survivin, down-regulating GSTs, and decreasing P-gp activity in JAR/MTX cells.

### 3.7 Stimulate the Body to Produce Specific Anti-tumor Immunity

Found that shikonin-induced apoptosis of ovarian cancer HO-8910 cells can promote the maturation of dendritic cells (DCs), which is the most functional antigen presenting cell found to date, and can induce specific anti-tumor immune response during maturation Li et al. (2014). In conclusion, shikonin can not only induce apoptosis but also stimulate specific anti-tumor immunity to achieve anti-tumor effect.

## 4 Structural Modification of Shikonin

**FIGURE 3:**
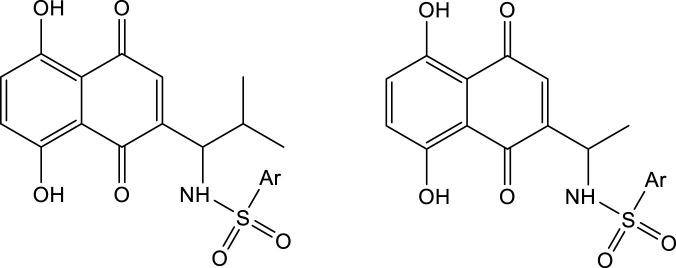
Synthesis of shikonin derivatives with arylsulfonamides side chains.

**FIGURE 4:**
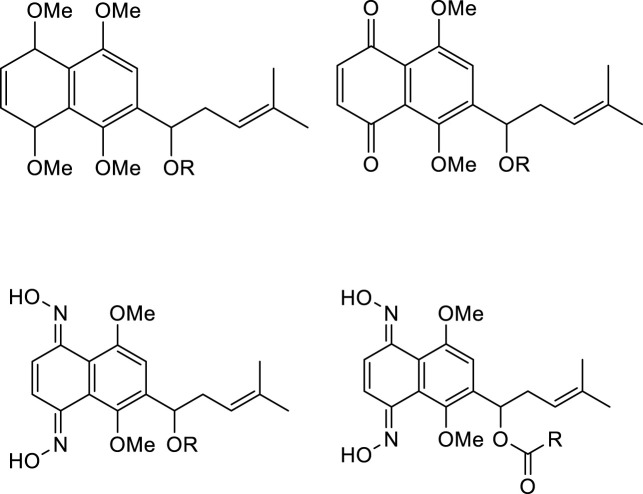
Synthesis of shikonin oxime derivatives.

**FIGURE 5:**
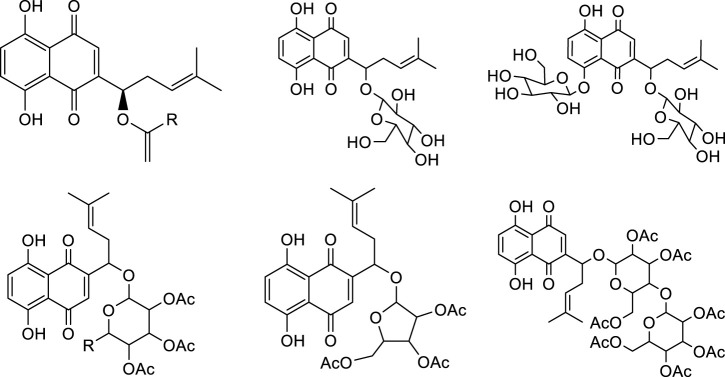
Synthesis of acetyl-β-glycosylshikonins.

As mentioned above, shikonin has received much attention because of its wide range of pharmacological activities. However, shikonin also has the common problem of anti-tumor drugs, that is, which has great toxic and side effects. Therefore, with the aim to make shikonin an antitumor drug for clinical, the structural modification of shikonin are necessary to either minimize its potential toxic side effects and/or to improve its potency. The parent naphthoquinone ring, branched-chain hydroxyl group and side-chain isohexene group of shikonin are all available for modification, and the isohexene side chain is the most important modification site. Synthesized analogues of benzenesulfonamide shikonin with good anti-human breast cancer MDA-MB-231 cells Wang et al. (2009). Synthesized acyl-β-glycosyl shikonin, which had obvious cell inhibition against adriamycin (ADR)-resistant cells (MCF-7/ADR, K562/ADR) and other drug-resistant tumor cells Su et al. (2010) (Figure 3). Obtained shikonin glycoside by structural modification with better water solubility and chemical stability Li et al. (2019). Meanwhile, the side chain and naphthoquinone ring were structurally modified. The researchers modified the naphthoquinone ring with hydroxyl oxime structure, which significantly improved the tumor targeting and reduced the toxic and side effects of the obtained shikonin oxime (Wang et al., 2014) (Figure 4). After continuous optimization of the structure, the obtained sulfur-containing shikonin oxime has stronger anti-tumor activity and lower toxic and side effects (Zhang et al., 2015; Huang et al., 2019) (Figure 5).

## 5 Discussion

Although many studies have proved that shikonin has the effect of anti-gynecological malignant tumors, there is still a long way to go before mature anti-tumor drugs are developed. Future work can be conducted mainly from the following aspects. 1) The anti-tumor studies of shikonin are mostly at the molecular biological level, and more animal models and comprehensive clinical trials are needed to further evaluate its effects. 2) Shikonin has certain toxic effects on normal cells, which is part of the reason why it cannot be used in clinic at present. At present, more than 100 naphthoquinone compounds have been synthesized artificially, so screening or synthesis of high efficiency and low toxicity derivatives is one of the effective measures to accelerate its application in clinical treatment. 3) Most malignant tumors require drug combination therapy. At present, studies have shown that shikonin or its derivatives have synergistic sensitization effect on malignant tumor-related chemotherapeutic drugs, but such studies are relatively few, and further studies in this regard can be carried out in the future. 4) Shikonin has unstable properties and is easy to react with surrounding substances and lead to inactivation. Therefore, the study of stable drug carriers is conducive to further research, such as the preparation of nanoemulsion, nanogels and liposome gel agents have certain application prospects. 5) The antitumor effect of shikonin is poor, so specific modification or preparation of targeting vector can be considered.
